# Bacterial Siderophores: Classification, Biosynthesis, Perspectives of Use in Agriculture

**DOI:** 10.3390/plants11223065

**Published:** 2022-11-12

**Authors:** Anna M. Timofeeva, Maria R. Galyamova, Sergey E. Sedykh

**Affiliations:** 1 SB RAS Institute of Chemical Biology and Fundamental Medicine, 630090 Novosibirsk, Russia; 2 Center for Entrepreneurial Initiatives, Novosibirsk State University, 630090 Novosibirsk, Russia; 3 Faculty of Natural Sciences, Novosibirsk State University, 630090 Novosibirsk, Russia

**Keywords:** siderophores, bacteria, biosynthesis, PGPB, agriculture, soil bacteria, soil microbiome, biofertilizers, rhizosphere, iron

## Abstract

Siderophores are synthesized and secreted by many bacteria, yeasts, fungi, and plants for Fe (III) chelation. A variety of plant-growth-promoting bacteria (PGPB) colonize the rhizosphere and contribute to iron assimilation by plants. These microorganisms possess mechanisms to produce Fe ions under iron-deficient conditions. Under appropriate conditions, they synthesize and release siderophores, thereby increasing and regulating iron bioavailability. This review focuses on various bacterial strains that positively affect plant growth and development through synthesizing siderophores. Here we discuss the diverse chemical nature of siderophores produced by plant root bacteria; the life cycle of siderophores, from their biosynthesis to the Fe–siderophore complex degradation; three mechanisms of siderophore biosynthesis in bacteria; the methods for analyzing siderophores and the siderophore-producing activity of bacteria and the methods for screening the siderophore-producing activity of bacterial colonies. Further analysis of biochemical, molecular–biological, and physiological features of siderophore synthesis by bacteria and their use by plants will allow one to create effective microbiological preparations for improving soil fertility and increasing plant biomass, which is highly relevant for sustainable agriculture.

## 1. Introduction

Fe ions are key components of various metabolic pathways in the cell. The Fe(II)/Fe(III) pair is involved in catalyzing a wide range of redox reactions and in electron transfer systems. Over a hundred metabolic enzymes have been described that possess iron-containing cofactors, such as Fe-S clusters or heme groups [1]. Fe is essential for many plant processes, including photosynthesis [2]: Fe is a part of photosystem I, cytochrome b6f complex, and photosystem II, with Fe ions also being required for chlorophyll synthesis and the general functioning of the photosynthetic apparatus [3]. In addition, some other proteins and protein complexes involved in electron transport during photosynthesis in chloroplasts and oxidative phosphorylation in mitochondria are Fe-dependent: non-heme Fe-S proteins (e.g., ferredoxin), heme proteins (e.g., catalase and peroxidase), and cytochromes [4]. Moreover, Fe serves as a cofactor in the synthesis of many plant hormones, such as ethylene and 1-aminocyclopropane-1-carboxylate [5]. Since free Fe(II) is rapidly oxidized to Fe(III), which is not bioavailable due to its low solubility, the amount of Fe for assimilation is extremely limited despite the abundance of Fe(III) in the Earth’s crust [1]. Fe deficiency in plants is a major economic issue that seriously impacts the quality and yield of crops [3].

A variety of bacteria referred to as plant-growth-promoting bacteria (PGPB) are capable of colonizing the rhizosphere and promoting Fe uptake by plants. These microorganisms can produce Fe ions under iron-deficient conditions. The PGPB of interest synthesize and release siderophores under appropriate conditions, thus increasing and regulating Fe bioavailability [6]. Siderophores are low-molecular-weight compounds (500–1500 Da) with a high affinity for Fe (III) (Kf > 10^30^). The affinity of siderophores for Fe is so high as to remove Fe from the molecules of Fe-binding proteins, for example, ferritin, transferrin, and lactoferrin [7,8]. Thus, the main function of siderophores is converting Fe bound to proteins or water-soluble compounds into a form accessible to microorganisms [9].

Siderophore-producing PGPB promote plant growth and improve host plant nutrition [10], but there are other benefits of PGPB for plants. For example, they can solubilize phosphates [11] and fix atmospheric nitrogen [12]. Given that both mineral phosphate and mineral (organic) nitrogen are required for siderophore synthesis, consortia of such PGPB are regarded as potential micro-fertilizers. Other PGPB may affect crop growth by reducing the impact of soil plant pathogens through the production of antimicrobial compounds and extracellular enzymes [13]. Siderophore-producing microbes generate numerous Fe-chelating compounds [14], thereby accelerating the physiological and biochemical processes of plants under unfavorable conditions [15,16]. Adding both Fe(III) and siderophores to the soil favors better plant growth compared to adding Fe(III) alone, as evidenced by the increase in plant weight [17].

With the world population constantly growing, the area of arable land decreasing, and the genetic potential of crops depleted, there is a strong necessity to introduce new agricultural technologies. Ensuring a high demand for high-nutritional-value food is possible when using low-impact agronomic solutions to increase plant resistance to adverse soil conditions [18,19]. In May 2020, the European Union announced the Farm to Fork (F2F) strategy, aimed at reducing the reliance on pesticides, antimicrobials, and excessive use of fertilizers. For the past few years, research has focused on novel agro-ecological approaches aimed at agro-biodiversity management [20].

Plant biostimulants are next-generation products likely to be effective for sustainable agriculture. Such plant biostimulants may combine chemical fertilizers with microorganisms and are therefore classified as microbial plant biostimulants [20]. Currently, there are studies aimed at selecting microorganisms with specific growth activity to improve the assimilation of nutrients in the case of their low availability, as well as at applying PGPB isolated from regions affected by salinization and desertification [20].

This review considers the main siderophore-producing PGPB, covers the classification of different types of siderophores produced by bacteria that are promising for agriculture, and describes the life cycle of siderophores from their biosynthesis synthesis in the bacterial cell to the release of Fe from the Fe–siderophore complex in the plant. Additionally, the methods for detecting siderophores and siderophore-producing bacteria are discussed.

## 2. Siderophore-Producing Bacteria and Their Potential Applications in Agriculture

Siderophore-producing bacteria have been described in 20 genera: *Azotobacter* [21,22], *Azospirillum* [23], *Bacillus* [24], *Dickeya* [25], *Enterobacter* [26], *Klebsiella* [27], *Kosakonia* [28], *Methylobacterium* [29], *Nocardia* [30], *Pantoea* [31], *Paenibacillus* [32], *Pseudomonas* [33], *Rhodococcus* [26], *Serratia* [34], *Streptomyces* [35] and others. The production of siderophores by bacteria is beneficial to plants and is considered to be a significant feature of PGPB [36], which can influence plant growth [37]. Siderophores produced by soil microorganisms supply Fe to plants and promote their growth. However, bacterial siderophores are responsible for limiting the development of some fungi and bacteria pathogenic to plants [38]. The biosynthesis of siderophores is not only characteristic of PGPB since some siderophore-producing bacteria are pathogenic to humans.

Several PGPB-producing siderophores also possess other plant-growth-promoting activities. A number of species of *Pantoea* produce siderophores: *P. diversa* [39], *P. agglomerans* [40], *P. eucalyptii* [41], *P. allii* [42], and *P. ananatis* [43]. The inoculation of soil *P. ananatis* has been shown to lead to the solubilization of phosphate and zinc and the production of siderophores and indole-3-acetic acid [44]. According to the whole-genome sequencing of *Pantoea* species, they have three clusters of genes homologous to clusters of different siderophore types (see Section 3 for details) [31].

PGPB that can simultaneously produce siderophores and possess nitrogen fixation have been described in a number of rhizobacteria: *Pantoea dispersa* and *P. cypripedii* AF1, *Enterobacter asburiae* [39], *Kosakonia arachidis* EF1 [41] and *K. radicincitans* BA1, and *Stenotrophomonas maltophilia* COA2 [28]. Such PGPB as *Variovorax paradoxus*, *Pseudomonas fluorescens* and *Bacillus megaterium* possess even more activities that are beneficial for plant growth: siderophore production, phosphate solubilization, exopolysaccharide production, indoleacetic acid production, and ACC deaminase activity under saline and normal conditions. Inoculation with these microorganisms was shown to positively affect the growth of cucumbers [45].

*B. subtilis* produces Fe-chelating compounds, resulting in improved wheat plant growth under drought conditions [46]. *B. subtilis* MF497446 and *P. koreensis* MG209738 were shown to produce siderophores and induce *Cephalosporium maydis* disease resistance in maize crops [47]. A strain of *Bacillus subtilis* 330-2 isolated from rapeseed was found to produce siderophores and significantly suppress fungal infections in vitro: *Rhizoctonia solani* AG1-IA, *Botrytis cinerea*, *Fusarium oxysporum*, *Alternaria alternata*, *Cochliobolus heterostrophus* and *Nigrospora oryzae*. This strain increased the growth of rice and corn seedlings [48]. *B. aryabhattai* MS3 was shown to increase rice yield by 60 and 43% in non-saline and saline (200 mM NaCl) conditions, respectively [15].

*Pseudomonas* sp. are widely presented in the rhizosphere of plants and stimulate growth by secreting enzymes and metabolites, solubilizing nutrients, and producing siderophores [49]. *P. chlororaphis*, *P. fluorescens*, *P. protegens*, *P. kilonensis P. putida*, *P. simiae and P. syringe* are used in agriculture to control plant diseases and increase yield [50,51,52]. *Pseudomonas* sp. GRP3 increased the chlorophyll level in siderophore-treated mung bean plants [53]. Under conditions of Fe deficiency, *Pseudomonas* sp. SP3 effectively stimulated the growth of apple tree rootstock and improved plant nutrition [54]. *Pseudomonas* sp. *IB-4* is capable of solubilizing phosphates, producing siderophores, and promoting plant growth [55].

*Azotobacter vinelandii* is a Gram-negative, free-living nitrogen-fixing bacterium possessing three nitrogenases with metal clusters. These nitrogenases are expressed in the presence of metal ions: Fe, Mo, and Va [56]. With all types of nitrogenases requiring Fe(III), *A. vinelandii* secretes siderophores with a potent ability to chelate Fe to ensure its uptake in Fe-limited environments [57]. The nitrogen-fixing bacterium *A. chroococcum* is capable of producing siderophores and positively affecting the growth of various crops under different soil types and climatic conditions. *A. chroococcum* AC1 and AC10 were shown to increase cotton biomass [22] and the content of soluble sugars in canola [58,59] and corn plants grown in saline soils [60]. In Argentina, *Azospirillum brasilense* Az39 isolated from wheat roots is recommended to be used in commercial preparations [61] due to its effective growth stimulation of this cereal [62].

An example of antagonistic activity is *Brevibacillus brevis* GZDF3 isolated from *Pinellia rhizosphere*, which is effective against *Candida albicans* fungal disease due to siderophore production [63].

A genome analysis of three cold-active strains of the Antarctic bacteria *Pseudomonas* sp. ANT_H12B, *Psychrobacter* sp. ANT_H59, and *Bacillus* sp. ANT_WA51 revealed the potential to stimulate plant growth through the secretion of various biomolecules, including siderophores. These bacteria stimulated alfalfa growth by increasing shoot length and biomass [64]. *Pseudomonas* sp. EMN2 isolated from the rhizosphere and inner parts of the roots of *Coffea arabica* plants was found to contain achromobactin– and aerobactin–siderophore receptors but to lack the genes responsible for producing these siderophores, indicating an interaction of this bacterium with other bacteria [65].

Unfortunately, many studies on the effect of siderophore-producing PGPB on plant growth and development revealed siderophore activity only by the chromium azurolsulfonate (CAS) test, which only allows one to establish the presence or absence of siderophore-producing activity. For more details on the structure of siderophores and examples of bacteria producing specific siderophores, the reader is referred to the next section.

## 3. Chemistry and Classification of Siderophores

All siderophores exhibit a higher affinity for Fe(III) than for Fe(II) without exception. Additionally, their affinity for Fe(III) is much higher than that of the other bivalent or trivalent metals. Moreover, siderophores can be involved in the uptake of several heavy metals from contaminated soils, which may be relevant for bioremediation [66].

The siderophore molecule usually has its iron atom coordinated with oxygen atoms, with the most common geometry being octahedral, allowing the six ligands to be arranged around the Fe center with minimal ligand repulsion. The octahedral field contributes to the formation of thermodynamically stable high-spin Fe(III) particles. Depending on the type of siderophore, the octahedral field can be distorted, and sometimes nitrogen or sulfur may be incorporated into the siderophore as coordinating atoms, with such siderophore variants having a lower affinity for Fe(III) [66]. The siderophore structure can have Fe(III) coordinated with such bidentate functional groups as hydroxamates, α-hydroxycarboxylates, and catecholates, as well as combinations of polydentate phenolates, nitrogen heterocycles, and carboxylates [67].

**Figure 1:**
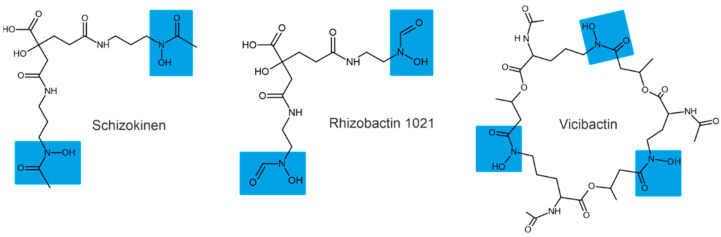
Examples of hydroxamate siderophores: shizokinen, rhizobactin, vicibactin. Hydroxamate functional groups are highlighted in blue.

**Figure 2:**
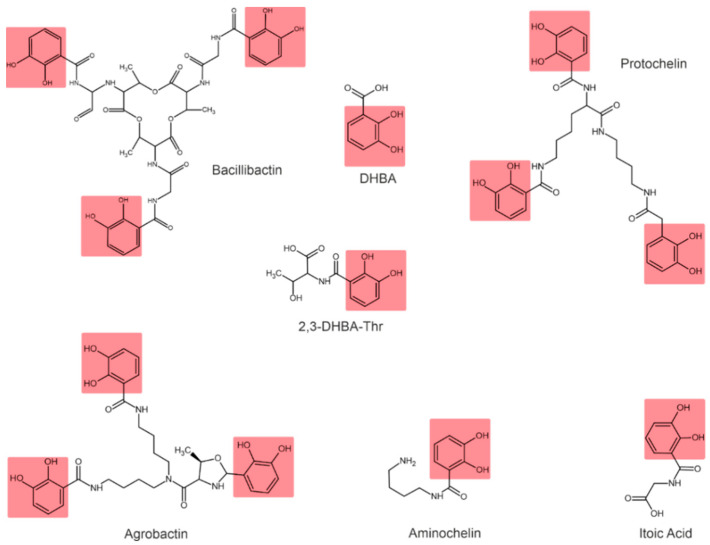
Examples of catecholate-type siderophores. Catecholate functional groups are highlighted in red.

**Figure 3:**
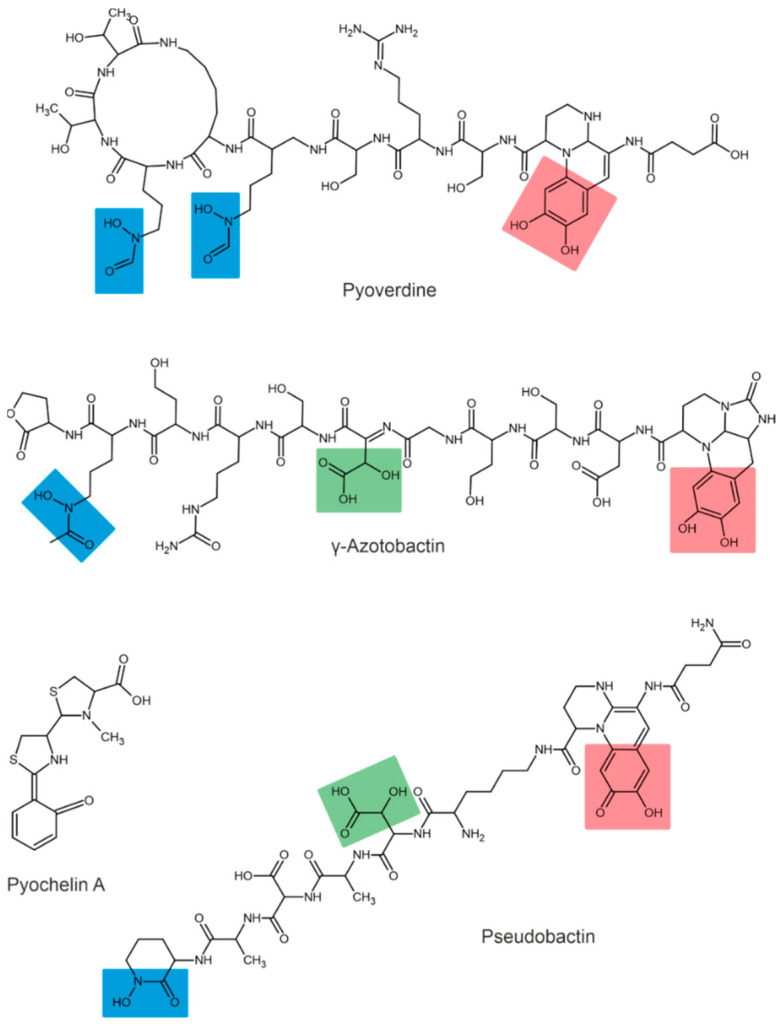
Examples of mixed-type siderophores. Hydroxamate groups are shown in blue, catecholate groups in red, and carboxylate groups in green.

Depending on their chemical nature, siderophores are classified into catecholates and phenolates, hydroxamates, carboxylates, and mixed-type siderophores [68,69]. Mixed-type siderophores correspond in their structure to two or three classes simultaneously. Therefore, they are treated as a separate class. The chemical structures of different classes of siderophores are shown in Figure 1, Figure 2 and Figure 3, with hydroxamate functional groups marked in blue, catecholate functional groups in red, and carboxylate functional groups in green. The specific features of siderophores of different classes will be discussed in the following subsections.

### 3.1. Hydroxamate Siderophores

Hydroxamate siderophores contain the structure C(=O)N-(OH)R, with R being an amino acid or its derivative containing two oxygen atoms forming a bidentate ligand with Fe ions. Each siderophore is capable of forming hexadentate ligands and octahedral complex compounds with Fe(III) ions [68]. When hydroxamate combines with Fe(III), its functional group loses a proton from the hydroxylamine group (-NOH) to form a bidentate ligand [70].

The bacterium *Bacillus megaterium* ATCC 19213 is known to produce two hydroxamate siderophores (shizokinen and N-deoxyshizokinen) under Fe-limited conditions [71]. In addition to the high affinity for Fe(III) ions, these siderophores are capable of chelating aluminum [72]. The *Rhizobium leguminosarum* IARI 917 is also known to produce the schizokinen siderophore [73]. The *Pantoea vagans* C9-1 produces hydroxamate-type desferioxamine-like siderophores [74]. Some strains of *Rhizobium radiobacter* are capable of producing hydroxamate-type siderophores [75]; for example, *Rhizobium meliloti* produces a siderophore called rhizobactin [76]. The *R. meliloti* 1021 produces a variant of rhizobactin called rhizobactin 1021 [77]. Vicibactin is a cyclic trihydroxamate siderophore and was found in *R. leguminosarum* and in *R. phaseoli* [78,79].

### 3.2. Catecholate Siderophores

In catecholate-type siderophores, the Fe(III) ion is bound to hydroxyl or catecholate groups. Upon chelation with Fe(III), a hexadentate–octahedral complex is formed, with two oxygen atoms from each catecholate group involved [80]. All catecholate siderophores are the derivatives of salicylic or 2,3-dihydroxybenzoic acid (2,3-DHBA) [23].

The catecholate-type siderophore, referred to as spirilobactin, is produced by *Azospirillum brasilense* in an Fe-depleted medium [81]. The *Azospirillum lipoferum* produces 2,3-dihydroxybenzoic acid (2,3-DHBA) and 3,5-DHBA conjugated to threonine and lysine [82], which also exhibit siderophore activity.

*Azotobacter vinelandii* is known to produce four catecholate siderophores: aminocholine, nitrocholine, protochelin, and 2,3-DHBA [57,68,83]. The *Rhizobium leguminosarum* IARI 102 also produces 2,3-DHBA conjugated to threonine (2,3-DHBA-Thr) [73].

*Bacillus subtilis* is characterized by the formation of 2,3-dihydroxylbenzoylglycine, also known as itoic acid [84]. A trimeric ester of this acid, referred to as bacillibactin, was described [85]. The production of bacillibactin was also described for *B. thuringiensis* [86]. The *Pantoea vagans* C9-1 produces the enterobactin-like catechol siderophore [87]. *Rhizobium radiobacter* produces a tricatecholate siderophore, which is called agrobactin [88,89].

### 3.3. Carboxylate and Mixed-Type Siderophores

Carboxylate-type siderophores bind to Fe via carboxyl and hydroxyl groups [80]. Carboxylate-type siderophores in PGPB have not been described in the literature. However, these siderophores are found among mixed-type siderophores.

In addition to the types mentioned above, some siderophores contain several Fe-chelating groups and are therefore classified as mixed-type siderophores.

The siderophores produced by fluorescent strains of *Pseudomonas* are pyoverdines [90,91]. All pyoverdines contain one quinoline chromophore, a peptide, and a dicarboxylic acid (or its corresponding amide) attached to the chromophore. The peptide is always the same in bacteria of the same strain but can differ across strains and species [92]. For example, three different pyoverdines, called pyoverdine, pyoverdine 0, and pyoverdine A (or ferribactin), have been isolated from *Pseudomonas fluorescens* [93]. Other bacteria of the genus *Pseudomonas*, such as *Pseudomonas syringae*, are also known to produce pyoverdine siderophores [94]. Additionally, *Pseudomonas aureofaciens* was reported to produce pyoverdine siderophores [95].

The first siderophore isolated from *Azotobacter vinelandii* was azotobactin [96], a pyoverdine-type siderophore. The pyoverdine structure of *A. vinelandii* was determined by the nuclear magnetic resonance method [97]. Chromophore has unique optical properties and specific absorption and fluorescence at 380 and 500 nm, respectively [56].

The structures of pseudobactin and pseudobactin A (distinguished by quinoline derivatives in the structure) were described for *Pseudomonas* B10 [98]. *Pseudomonas fluorescens* produces several other siderophores, such as enantio-pyochelin [99], quinolobactin [100], ornicorrugatin and pseudomonins [101]. The *Pantoea eucalypti* M91 is capable of producing pyoverdine-like and pyochelin-like siderophores in alkaline media [10].

It is worth noting that siderophores exhibit antifungal properties. One of the most studied siderophores with direct antifungal properties is pyoverdine produced by *Pseudomonas* spp., which contributes to the suppression of pathogen development by increasing competition for Fe: fungal siderophores generally have a lower affinity for Fe(III) than bacterial siderophores [53]. For example, *P. fluorescens* WCS374r (Psb374) was shown to be necessary for inducing siderophore-mediated resistance in rice when infected with *Magnaporthe oryzae*. The inoculation of soil with a mutant WCS374r strain deficient in pseudobactin and the subsequent infection of rice leaves with *M. oryzae* after 4–5 days demonstrated no suppression of siderophore–mutant bacteria disease in rice compared with the wild-type *P. fluorescens* [102].

Pyoverdins are supposed to be involved in the biological control of phytopathogenic microorganisms in the rhizosphere, as they are known to form stable complexes with soil Fe, making this essential element unavailable for consumption by harmful rhizosphere microorganisms [103].

Table 1 presents the major siderophore-producing PGPB with an established siderophore structure.

## 4. Biosynthesis of Siderophores

Siderophore biosynthesis in bacteria is performed by several enzymes: non-ribosomal peptide synthetase (NRPS), polyketide synthase (PKS), and NRPS-independent siderophore synthetase (NIS) [110].

### 4.1. Siderophore Biosynthesis by NRPS

Siderophores synthesized by NRPS are primarily composed of amino acids, including non-proteinogenic amino acids, linked together by peptide bonds [111,112]. NRPS are large multi-domain and multi-enzyme complexes, with each subunit responsible for attaching one amino acid to a growing peptide chain, including non-proteinogenic amino acids and hydroxy acids [113]. The standard NRPS architecture comprises modular sequences of adenylation (A), condensation (C), peptidyl carrier protein (P) and thioesterase (T), as well as other specific functional domains including epimerization (E), oxidation (Ox), methylation (Mt) and cyclization (Cy) [113,114].

**Figure 4:**
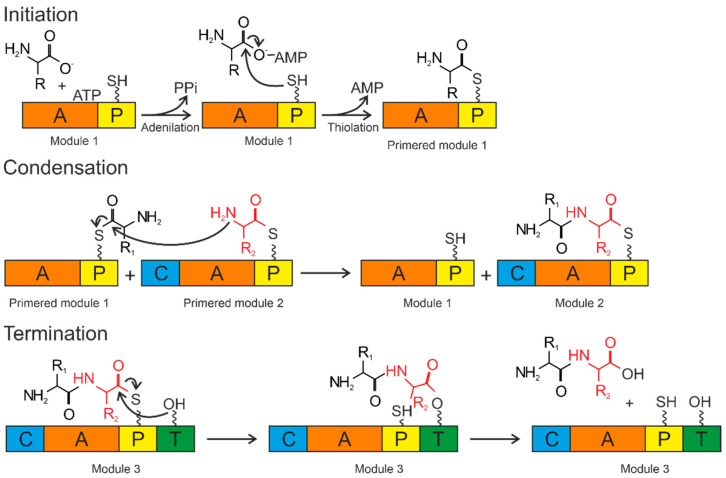
Biosynthesis by NRPS occurs in three steps: initiation, condensation, and termination. The A domain activates the amino acid and attaches it to the P domain (initiation). The condensation reaction occurs between the amino acids bound to the primed modules to form a peptide bond, resulting in the elongation of the peptide. Termination and release of the mature peptide occur by hydrolysis in the thioesterase domain after the product is transferred to the conserved serine residue. Orange—adenylation domain (A); yellow—peptidyl domain (P); blue—condensation domain (C); green—thioesterase domain (T).

The A domains are called the “gatekeepers” of the NRPS assembly line due to selectively activating and incorporating the corresponding amino acids into the growing peptide chain [115]. The amino acid is activated by conversion to the aminoacyl–AMP domain by the A domain. Then, it is covalently attached (with loss of AMP) to the P domain (Figure 4).

The P domain acts as a binding system for the growing peptide chain. The P domain contains a post-translational modification by coenzyme A over the conserved serine residue. As a result, a swinging 4’-phosphopantetheinyl (P_pant_) shoulder is formed. The P_pant_ P domain thiol performs a nucleophilic attack on the carboxyl group of aminoacyl–AMP, removing AMP and forming an aminoacyl–thioether bond (initiation). After priming, the thioether bond on the P domain transfers the amino acid sequence to the next domains. The formation of the first peptide bond or peptide elongation is catalyzed by amino acid residues of the two primed modules within the C domain. The thioether bond is broken as the peptide bond is formed, resulting in a peptide elongation. These reactions occur until peptide synthesis is complete. Chain elongation is terminated by the thioesterase (T) domain. The T domain removes the entire chain from the last domain. The peptide is transferred from the P domain to the conserved serine residue of the T domain, forming an amino ether and allowing the hydrolysis and release of the mature peptide [116].

The secondary metabolites generated by NRPS are more than just short peptides. Siderophores contain metal-chelating thiazoline or oxazoline rings added by Cy/Ox domains to cysteine and serine residues. Additionally, the C-glycosylation of enterobactin occurs, catalyzed by glycosyltransferase IroB, during the synthesis of salmohelin [117]. Most bacteria have their genes encoding NRPS and aryl acid synthesis enzymes directly regulated by iron via the repressor Fur.

In some cases, NRPS are partially involved in the generation of hydroxamate and carboxylate siderophores by synthesizing a peptide backbone to which iron-coordinating residues are attached. This has been shown for *S. coelicolor* celichelin [118,119], exochelins of nonpathogenic mycobacteria [120,121] and ferricchromes/ferricercins from various fungi [122,123,124].

### 4.2. Siderophore Biosynthesis by Polyketide Synthases

**Figure 5:**
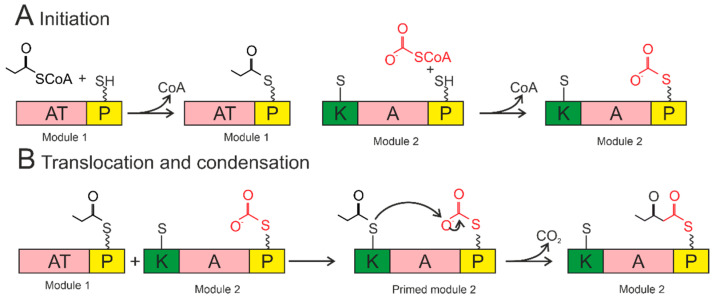
Siderophore biosynthesis by polyketide synthase. Acyltransferase domains load acyl groups on the P domains of Module 1. The acyl group from the loading module is transferred to the ketosynthase domain of Module 1, thereby initiating the module (bottom left). The ketosynthase domain catalyzes the condensation reaction. The growing chain is transferred to the ketosynthase domain of the next module to continue synthesis. AT—acyltransferase; P—acyl carrier protein; K—ketosynthase. The wavy line marks the post-translational modification of P_pant_. The short straight line in the KS domain denotes the cysteine residue of the active center.

Some siderophores are synthesized by polyketide synthases. The PKS module includes a ketosynthase domain, an acyltransferase domain, and a carrier domain (the schemes a presented in Figure 5). The initiation module is covalently attached to the carrier domain by the acyltransferase domain, releasing CoA (Figure 5A). The acyl chain is transferred from the P domain of the loading module to the cysteine in the ketosynthase domain (Figure 5B). The ketosynthase domain catalyzes a condensation reaction whereby the growing chain attaches to the carrier domain of the first module and can be moved to the ketosynthase domain of the next module. As a result, this conveyor mechanism results in various domains being incorporated into each module, allowing different functions to be added. The following modifications are possible: ketoreductases, dehydratases, methyltransferases, and oxidases. The thioesterase domain removes the entire chain from the final carrier domain by reduction, hydrolysis, or sometimes cyclization [125,126]. The P domains in PKS, as in NRPS, provide cotyledon and amino acid incorporation into the siderophore chain skeletons.

### 4.3. Siderophore Biosynthesis by NIS Synthetase

Some siderophores are synthesized not by NRSP or PKS [67], but by NRPS-independent siderophore synthetases [127]. NIS synthetases form siderophores containing citric acid, α-ketoglutarate, or succinic acid. The NIS synthetase contains an acyladenylation domain that forms, for example, citrate–AMP, providing an energy-rich bond for the condensation reaction with an amino acid or polyamine. Siderophores constructed with NIS include aerobactin, achromobactin (*Pseudomonas syringae*), desferioxamine (*Streptomyces griseus*), baumannoferrin (*Acinetobacter baumannii*) [128], putrefactins (*Shewanella putrefaciens*).

## 5. Secretion of Siderophores into the Environment, Transport of Fe–Siderophore Complexes into the Cell

After the biosynthesis, apo-siderophores are secreted into the medium. Several different secretion systems have been identified, including transporters from the major facilitator superfamily (MFS) and efflux pumps from the resistance, nodulation, and cell division (RND) superfamily [129].

**Figure 6:**
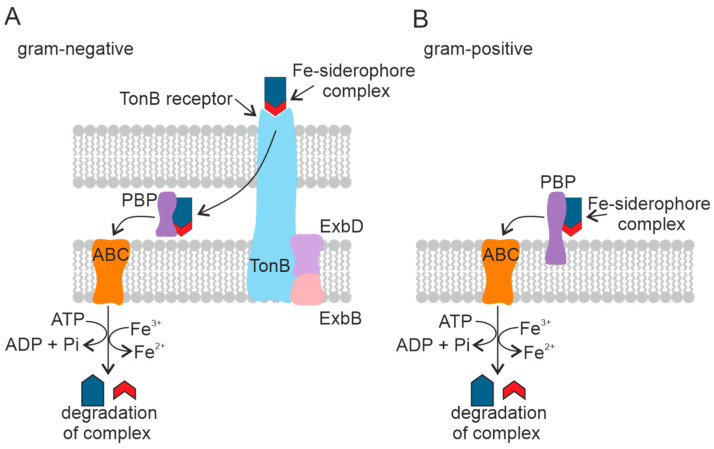
Mechanism of transport of the Fe–siderophore complex into the cell in Gram-negative (**A**) and Gram-positive (**B**) PGPB. Periplasmic binding protein (PBP), ATP-binding cassette transporter (ABC). The TonB-dependent receptor is a complex of three proteins.

Many NRPS-based siderophore gene clusters contain a gene encoding the MFS transporter, a member of the broad substrate transporter group [130]. The MFS protein *YmfE* involved in the secretion of the siderophore bacillibactin was identified in *B. subtilis* [131]. The mutant strain deficient in the *YmfE* gene was shown not to grow in Fe-deficient medium. The RND superfamily is a group of proton antiporters particularly common among Gram-negative bacteria [132]. See Figure 6A.

Fe–siderophore complexes bind to specific outer membrane receptors with high affinity (Kd ~ 0.1 µM). In contrast to porins, which use passive diffusion to absorb dissolved substances, outer membrane receptors actively pump siderophores into the periplasm against the concentration gradient using an energy-dependent transport mechanism. Several siderophore-mediated Fe-uptake pathways consisting of an outer membrane receptor, a periplasmic binding protein, and a complex of one or two cytoplasmic membrane proteins with an associated ATP-binding cassette (ABC) forming altogether a carrier have been described in Gram-negative bacteria. Most bacteria have separate systems, each specific for one siderophore. Bacteria are known to be able to use siderophores secreted by coexisting microbes [133]. Despite different structures and properties, all siderophores have a peptide backbone that interacts with outer membrane receptors present on the cell surface [134].

There are different systems used by microorganisms to transport Fe–siderophore complexes. The transport systems differ between Gram-positive bacteria and Gram-negative bacteria. In Gram-negative bacteria, the pathway for the Fe–siderophore complex to enter the cell is arranged so that Fe chelates are transported across the outer membrane, the periplasm, and the cytoplasmic membrane by discrete transporters. The proteins required for each transport step are localized in different cell membrane compartments and have specific energy requirements [133].

The transport of siderophores across the outer cell membrane of Gram-negative bacteria is mediated by a complex of three transmembrane proteins: TonB, ExbD, and ExbB [133]. This family of outer membrane transport proteins is called TonB-dependent receptors. TonB-dependent receptors consist of five copies of ExbB, two copies of ExbD, and TonB [135]. The Ton complex proteins are embedded in the cytoplasmic membrane and penetrate the periplasm [136]. TonB-dependent receptors recognize Fe(III)–siderophore complexes on the cell surface [137]. The Fe(III)–siderophore complex is then bound to the periplasmic binding protein [138], which accompanies the complex to the cytoplasmic membrane and is released into the periplasmic space [139]. The Fe–siderophore complex is transported from the periplasm into the cytoplasm through the inner membrane by the ABC and reaches the cytoplasm as an Fe(II) ion [140].

Gram-positive bacteria lack the outer membrane and the corresponding receptors. The Fe(III)–siderophore complexes are bound by periplasmic siderophore-binding proteins fixed on the plasma membrane [141], followed by Fe(III)–siderophore complexes being transported into the cytoplasm by the ABC transport system in the same manner as in Gram-negative bacteria [142]. See Figure 6B.

### 5.1. The Fate of the Fe–Siderophore Complex in the Cell

Inside the bacterial cell, Fe ions are released from the Fe–siderophore complex and become available for metabolic processes. Two main mechanisms of Fe release have been described. The first involves the reduction of siderophore-bound Fe(III) to Fe(II) followed by the spontaneous release or competitive binding of reduced ions [66]. To date, two families of proteins, siderophore-interacting protein (SIP) and Fe–siderophore reductase (FSR), involved in this process have been identified.

The second pathway of Fe release uses specialized enzymes that hydrolyze the Fe–siderophore complex and destabilize it [1]. Examples of such enzymes are esterases of the α/β-hydrolase family of enzymes [143]. Due to causing the siderophore backbone destruction, the hydrolytic release of Fe is more costly for the cell than Fe siderophore reduction, mostly allowing the siderophore to be reused.

The fate of Fe after its release in the bacterial cell may be to bind to spare proteins such as ferritins, bacterioferritins, and ferrochelatin [1].

### 5.2. The Fate of the Fe–Siderophore Complex outside the Bacterial Cell

Bacterial siderophores are known to provide plants with Fe and promote their growth when Fe bioavailability is low. The exact mechanisms of these processes have not been established, but two possible ways for plants to obtain Fe from microbial siderophores have been proposed. The first mechanism suggests that bacterial siderophores with a high redox potential can be reduced to give Fe(II) back to the plant transport system. It is proposed that, according to this mechanism, Fe(III)–siderophores from bacteria are first transported to the plant root apoplast, where siderophore reduction occurs. Thus, Fe(II) is captured by the apoplast, possibly leading to a high local concentration of Fe in the root. The second mechanism is for the bacterial siderophores to chelate Fe from the soil and perform ligand exchange with phyto-siderophores [144]. The mechanisms described are theoretical and have not yet been confirmed experimentally.

In nature, most bacterial producers of siderophores are related to the rhizosphere of plants. For this reason, siderophore concentrations are highest in the rhizosphere, where 0.1 μM to mM concentrations of siderophores have been demonstrated [145]. The concentration of siderophores decreases significantly in the soil outside the rhizosphere, where it can be as low as 10 nM [146]. In the soil, siderophores are adsorbed on clay minerals and organic matter [147].

In the environment, siderophores can undergo abiotic degradation through hydrolysis and/or oxidation. Siderophores with hydroxamate groups can hydrolyze to form hydroxylamine groups, during which Fe(III) is reduced to Fe(II) [148].

Exposure to sunlight can also stimulate the degradation of siderophores. For example, hydroxycarboxylates in complex with Fe are photoreactive, and catecholates, on the contrary, are photoreactive only in the absence of Fe; hydroxamates are not photoreactive [149].

Free siderophores inevitably interact with various organisms and are absorbed by bacteria and plants [150]. The ability of some bacteria to use siderophores as a source of carbon and nitrogen was described [151].

**Figure 7:**
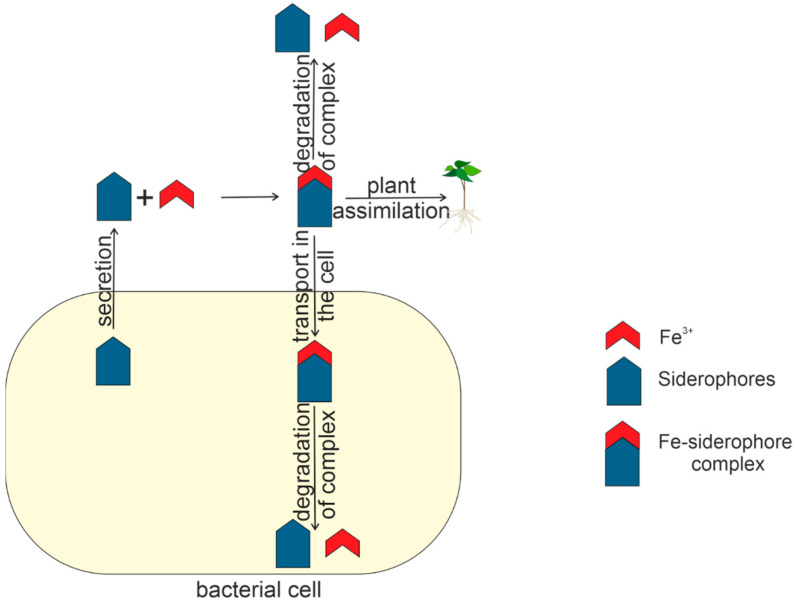
The life cycle of siderophore in and outside the bacterial cell.

Figure 7 shows the life cycle of siderophores within and outside the bacterial cell.

## 6. Application of Siderophores to Control Phytopathogens: Use of Siderophores in Soils Contaminated with Heavy Metals

Siderophores play an important role in the biological mechanism of controlling some phytopathogens. Since siderophores firmly bind Fe and reduce its bioavailability to plant pathogens, they can facilitate the destruction of phytopathogens [144,152]. For example, pyoverdine siderophores produced by *Pseudomonas chlororaphis* YL-1 were shown to possess extensive antimicrobial activity against phytopathogens [153]. In addition, *Pseudomonas orientalis* F9 was shown to exhibit antagonistic properties toward phytopathogens and contain genes of pyoverdine biosynthesis. The antagonistic effect of *Pseudomonas syringae* pyoverdins on *Caenorhabditis elegans* was demonstrated [154]. In addition to pseudomonads, siderophores produced by *Bacillus subtilis* also are of crucial importance in the biocontrol of *Fusarium oxysporum*, leading to fusarium wilt in pepper [155]. To sum up, siderophores can be regarded as a potential biological control agent against several phytopathogens.

Metals other than Fe are also capable of stimulating or inhibiting siderophore production in a number of bacteria, even in the presence of high concentrations of Fe. For example, the presence of Mo in *Azotobacter vinelandii* media regulates the production of azotochelin, a catecholate siderophore that can chelate this metal [156]. High concentrations of Al were shown to enhance the production of schizokinene and N-deoxyschizokinene (both hydroxamate siderophores) in Fe-limited cultures but not in cultures of *Bacillus megaterium* with high Fe content [72].

The fact that toxic metals were observed to induce the production of some siderophores suggests that these siderophore chelators may play an important role in the resistance of bacteria to heavy metals. Toxic metals enter the periplasm of Gram-negative bacteria mainly by diffusion through porins [157]. Thus, the binding of metals to siderophores in the extracellular medium decreases the concentration of free metals, thus probably reducing diffusion, since the molecular weight of the formed siderophore–metal complex is too large for its diffusion through porins and, consequently, it affects their toxicity.

Siderophores can modify the degree of oxidation of heavy metals such as Cd, Cu, Ni, Pb, Zn and Th, U, and Pu, making them less toxic [157]. Siderophores also bind various toxic Cr, Cu, Pb, Cu, V, and Al, with the binding ability of siderophores to Fe being greater than to other heavy metals [158,159]. Thus, the ability of siderophore to detoxify and bind toxic heavy metals plays a prominent role in plant growth in soil contaminated with heavy metals.

The bacterial strain *P. fluorescence* produces the pyoverdine-type siderophore, which increases mobility and reduces the toxicity of heavy metals in uranium mines [160]. Two species of *Providencia* sp. (TCR05) and *Proteus mirabilis* (TCR20) were shown to lower Cr toxicity by reducing Cr(VI) to Cr(III) in contaminated soils [161]. PGPB rhizobacteria *Streptomyces tenae* F4 phytormediate Cd and enhance the uptake of other metals in soils contaminated with heavy metals [162]. The *Rhizobium* strains promote Cu uptake, while the *Pseudomonas* strain promotes Cu and Fe uptake by *Phaseolus vulgaris* plants [163], and *P. acidiscabies* secretes the hydroxamate types of siderophores responsible for Ni and Fe dissolution and absorption by *Vigna unguiculata* plants under nickel stress [164]. The symbiotic association of *Kluyvera ascorbata* and plants decreases heavy metal toxicity [165] and suppresses phytopathogens [166].

## 7. Methods for Siderophore Detection and Characterization

The characterization of siderophore-producing activity is usually performed by a combination of several methods. At first, bacterial colonies are screened on solid agarized media to determine whether they are capable of producing siderophores. Next, the type of siderophore can be determined on Petri dishes: hydroxamate, catecholate, or carboxylate.

For a more detailed examination, siderophores can be identified by HPLC, with NMR and mass spectrometry also used to establish the structure. Gene expression analysis is used to establish the changes in the transcriptional activity of genes depending on the Fe concentration in the medium. This method allows one to establish the genes responsible for the siderophore-producing activity.

### 7.1. Method for Determining the Presence of Siderophore-Producing Activity with Chromium Azurolsulfonate

**Figure 8:**
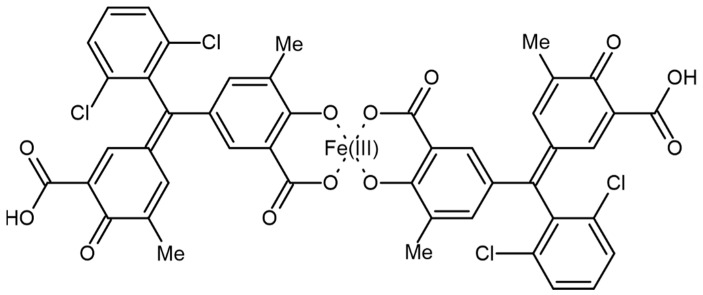
Structure of chromazurol S complex with Fe(III).

The ability of microorganisms to produce siderophores is usually determined by the chromium azurolsulfonate (CAS) assay [167]. The possible structure of Fe(III) and CAS is presented in Figure 8 [168].

The chromazurol S × Fe(III) × hexadecyltrimethylammonium bromide (HDTMA) ternary complex is an indicator: when the potent chelator removes Fe from the dye, its color changes from blue to orange; see Equation (1) [167].

(1) $$FeDye^{3-\lambda }+L^{\kappa -}\to FeL^{3-\kappa }+Dye^{\lambda -}$$

The reaction of Fe(III) with chromazurol S. *L* is the siderophore under study.

This method is highly sensitive and allows one to detect siderophores on the supernatant of the culture liquid. Siderophores secreted by selected bacterial cultures can be quantitatively analyzed by growing the bacterial cultures in the Modi medium. In this case, the amount of siderophore is measured spectrophotometrically [167].

Solid agar medium containing CAS is also used for the qualitative determination of siderophore production. Orange halos around the colonies on blue agar indicate the excretion of siderophores.

Both methods require the CAS reagent to be prepared according to Schwyn and Neilands [167]: 121 mg of CAS are dissolved in 100 mL of distilled water, and 20 mL of 1 mM FeCl_3_·6H_2_O in 10 mM HCl are added to the solution. To the resulting solution, 20 mL of HDTMA solution (729 mg HDTMA in 400 mL of distilled water) are added under stirring. The CAS–HDTMA solution is sterilized before further use.

The siderophore content is quantified in the supernatant of a bacterial culture grown in the LB medium. The supernatant of 0.5 mL is mixed with 0.5 mL of CAS reagent, and the optical density is measured after 20 min at 630 nm. The amount of siderophores produced by the strains is measured in percent siderophore units (psu), which are calculated using the formula [169]:

$$Siderophore\;Unit\;\left(\%\right)=\frac{\left(A_{r}-A_{s}\right)}{A_{s}}\times 100$$

with A_r_ being the optical density at A_630_ nm (CAS analysis solution mixed with an equal volume of unseeded medium) and A_s_ being the optical density of the sample at 630 nm (CAS analysis solution + supernatant) [167].

### 7.2. Identification of Catechin and Hydroxamate Groups

The technique described above does not determine the type of siderophore. The Arnow [170], Csáky [171], and Shenker [172] tests are used to analyze the type of siderophore contained in the nutrient medium. The Arnow test detects the presence of catechin groups, the Csáky test is used to detect hydroxamic groups, and the Shenker test is used to detect carboxylates.

The Arnow method is based on the reaction between the catechol and the nitrite–molybdate reagent in an acidic medium with the formation of a yellow color. In an alkaline medium, the color changes to an intense orange–red color. The protocol is the following: mix 1.0 mL of supernatant with 1.0 mL of 0.5 M HCl, add 1.0 mL of sodium nitrite and molybdate (10 g sodium nitrite and 10 g sodium molybdate dissolved in 100 mL of deionized water) and 1.0 mL of 1 M NaOH. The color development occurs at room temperature for 5 min. If a catecholate siderophore is present, the solution stains orange–red. The intensity of the staining depends on the amount of catechol present [170].

The Csáky test detects hydroxamate-type siderophores by the formation of a stained complex [171]. The protocol is the following: 1.0 mL of 6 N H2SO4 is added to 1.0 mL of supernatant, incubated for 30 min at 130 °C. Add 3.0 mL of sodium acetate (350 g/L) and 1.0 mL of sulfanilic acid (10 g/L in 30% acetic acid *v*/*v*), followed by 0.5 mL of iodine solution (13 g/L in glacial acetic acid). After 3–5 min, the excess iodine is neutralized by adding 1.0 mL of 20 g/l sodium arsenite. Finally, 1.0 mL of α-naphthylamine solution (3 g/l in 30% acetic acid *v*/*v*) is added and left to develop color for 20–30 min. Dark pink staining indicates the presence of hydroxamates in the solution.

### 7.3. HPLC, NMR, and Mass-Spectroscopy

Fractions are lyophilized and reduced with D_2_O (vibrioferrin, aminohelin) or deuterated MeOH to obtain 1H-NMR and correlation spectroscopy. Siderophores are quantified by 1H-NMR with the addition of an internal sodium benzoate standard for vibrioferrin or by UV–visible emission using the literature extinction coefficients for acidified solutions of DHBA, aminohelin, nitrohelin, protochelin, and azotobactin [173,174]. Petrobactin was isolated by the UHPLC method on a C18 sorbent [86].

Hydroxamates are isolated from liquid cultures by benzyl alcohol extraction and purified by gel filtration and HPLC. Hydroxamates have a characteristic absorption maximum at 420–423 nm, which does not shift between pH 3.0 and 9.0. By combining cyclic voltammetry of Fe complexes with hydroxamate with mass spectra, the molecular weights of the compounds can be determined. To prove the presence of hydroxamic acids, reductive hydrolysis in 57% hydrogen iodide acid is used, resulting in the formation of ornithine, which is determined by tandem gas chromatography and mass spectrometry [175].

Azotobactin synthesized by *A. vinelandii* can be determined spectrophotometrically. The optical density of azotobactin not bound to metal ions is very sensitive to pH: at pH 7.0, azotobactin has two peaks, at 380 and 420 nm, with the peak at 420 nm decreasing with decreasing pH, whereas at pH 7.5, there is one peak at 420 nm. On the other hand, the siderophore bound to metal ions shows one peak at 380 nm at all pH [56]. Given the differences in the siderophore absorption profile, one can determine azotobactin in complex with Fe and individually.

A recent advance in siderophore research is using electrospray and high-resolution liquid chromatography ionization mass spectrometry (HR–LC–MS) techniques that exploit the characteristic isotope structure of ^54^Fe–^56^Fe associated with organic chelates [176,177,178]. Data analysis techniques have been developed to filter out the relevant isotopic structures associated with Fe complexes, even at low contents and in highly complex matrices, and to detect relevant aposiderophores [176]. The species thus identified can then be characterized by analyzing the tandem MS and MS/MS spectra and additional UV spectroscopy and NMR data.

Siderophores in supernatant extracts are analyzed by capillary liquid chromatography followed by ESI-MS or ESI-MS/MS detection. Liquid chromatography is performed using C18 columns, and elution is performed with a mobile phase containing 5% *v*/*v* acetonitrile in 11 mM ammonium formate at pH 4.0. A mass spectrometer is used in ESI positive scan mode between *m*/*z* 550 and 750 amu [179].

## 8. Conclusions

Plants interact with beneficial and pathogenic microorganisms that can produce siderophores. The analysis of the mechanisms of siderophore synthesis and their effect on plant growth and development are essential for the development of new strategies for rational farming.

Despite considerable scientific interest in siderophore-producing bacteria as new biofertilizers, there is currently no understanding of the relationship between the nature of siderophores and their effect on plant growth and development. Nor is there an understanding of the exact mechanism by which plants assimilate Fe with the help of bacterial siderophores.

Further analysis of biochemical mechanisms and molecular biological features of siderophore biosynthesis and its physiological role is required to find new efficient combinations of rhizospheric PGPB to obtain consortia leading to a comprehensive increase in plant productivity.

## Figures and Tables

**Table 1:** Major siderophore-producing PGPB for which the siderophore structure has been established.

Genus	Strain	Gram	Siderophore		Reference
			Name	Type	
*Azospirillum*	*Azospirillum brasilense*	Negative	Spirilobactin	Catechol	[81]
	*Azospirillum lipoferum*	Negative	2,3-DHB, 3,5-DHB-threonine, 3,5-DHB-lysine	Catechol	[82]
*Azotobacter*	*Azotobacter vinelandii*	Negative	Aminochelin, Azotochelin, Protochelin, 2,3-DHB	Catechol	[68,104,105]
	*Azotobacter vinelandii*	Negative	Azotobactin	Mixed	[56,96]
		Negative	Vibrioferrin	Mixed	[21]
*Bacillus*	*Bacillus megaterium*	Positive	Schizokinen, N-schizokinen, N-schizokinen-A	Hydroxamate	[71,106]
	*Bacillus subtilis*, *Bacillus thuringiensis*	Positive	Itoic acid, Bacillobactin	Catechol	[85,107]
*Pantoea*	*Pantoea vagans C9-1*	Negative	Enterobactin-like	Catechol	[74]
		Negative	Desferrioxamine-like	Hydroxamate	[74]
	*Pantoea eucalypti M91*	Negative	Pyoverdine-like, Pyochelin-like	Mixed	[10]
*Pseudomonas*	*Pseudomonas B10*	Negative	Pseudobactin(s)	Mixed	[98]
	*Pseudomonas fluorescens*, *Pseudomonas aeruginosa*, *Pseudomonas syringae*, *Pseudomonas aureofaciens*	Negative	Pyoverdine(s)	Mixed	[94,95,108,109]
	*Pseudomonas fluorescens*	Negative	Ferribactin	Mixed	[93]
*Rhizobium*	*Rhizobium radiobacter*	Negative	Agrobactin	Catechol	[88,89]
	*R. leguminosarum*, *R. phaseoli*	Negative	Vicibactin	Hydroxamate	[78,79]
	*Rhizobium leguminosarum*	Negative	Schizokinen	Hydroxamate	[73]
		Negative	2,3-DHB-threonine	Catechol	[73]
	*Rhizobium meliloti*	Negative	Rhizobactin	Catechol	[77]

## Data Availability

Not applicable.
